# Integrin αvβ1 facilitates ACE2-mediated entry of SARS-CoV-2

**DOI:** 10.1016/j.virusres.2023.199251

**Published:** 2023-11-02

**Authors:** Zeqiong Cai, Han Bai, Doudou Ren, Biyun Xue, Yijia Liu, Tian Gong, Xuan Zhang, Peng Zhang, Junsheng Zhu, Binyin Shi, Chengsheng Zhang

**Affiliations:** a The MED-X Institute, The First Affiliated Hospital of Xi'an Jiaotong University, Building 21, Western China Science and Technology Innovation Harbor, Xi'an 710000, China; b Precision Medicine Center, The First Affiliated Hospital of Xi'an Jiaotong University, 277 Yanta West Road, Xi'an 710061, China; c Center for Molecular Diagnosis and Precision Medicine, The First Affiliated Hospital of Nanchang University, 17 Yongwai Zhengjie, Nanchang 330006, China; d Department of Clinical Laboratory, The First Affiliated Hospital of Nanchang University, 17 Yongwai Zhengjie, Nanchang 330006, China; e Department of Endocrinology, The First Affiliated Hospital of Xi'an Jiaotong University, 277 Yanta West Road, Xi'an 710061, China; f Department of Medical Genetics, The First Affiliated Hospital of Nanchang University, 17 Yongwai Zhengjie, Nanchang 330006, China

**Keywords:** COVID-19, SARS-CoV-2, ACE2, Integrin αvβ1, Viral entry

## Abstract

Integrins have been suggested to be involved in SARS-CoV-2 infection, but the underlying mechanisms remain largely unclear. This study aimed to investigate how integrins facilitate the ACE2-mediated cellular entry of SARS-CoV-2. We first tested the susceptibility of a panel of human cell lines to SARS-CoV-2 infection using the spike protein pseudotyped virus assay and examined the expression levels of integrins in these cell lines by qPCR, western blot and flow cytometry. We found that integrin αvβ1 was highly enriched in the SARS-CoV-2 susceptible cell lines. Additional studies demonstrated that RGD (403–405)→AAA mutant was defective in binding to integrin αvβ1 compared to its wild type counterpart, and anti-αvβ1 integrin antibodies significantly inhibited the entry of SARS-CoV-2 into the cells. Further studies using mouse NIH3T3 cells expressing human ACE2, integrin αv, integrin β1, and/or integrin αvβ1 suggest that integrin αvβ1 was unable to function as an independent receptor but could significantly facilitate the cellular entry of SASR-CoV-2. Finally, we observed that the Omicron exhibited a significant increase in the ACE2-mediated viral entry. Our findings may enhance our understanding of the pathogenesis of SARS-CoV-2 infection and offer potential therapeutic target for COVID-19.

## Highlights

• Integrin αvβ1 was highly enriched in the SARS-CoV-2 susceptible cell lines.
• Integrin αvβ1 significantly facilitated the entry of SASR-CoV-2 into cells.
• Anti-αvβ1 integrin antibodies could inhibit the entry of SARS-CoV-2 into cells.
• The RBD with RGD→AAA mutation was defective in binding to integrin αvβ1.
• The viruses bearing the RGD→AAA mutation exhibited much lower entry efficiency.

## 1 Introduction

The spike glycoprotein (S) of SARS-CoV-2 binds to the cellular receptor and mediate membrane fusion for virus entry into host cells (Ke et al., 2020). While human angiotensin-converting enzyme 2 (ACE2) is the major receptor for SARS-CoV-2 (Scialo et al., 2020), several alternative receptors for ACE2 have been identified, such as neuropilin-1, CD147, and AXL (Andrews et al., 2021; Caccuri et al., 2021; Cantuti-Castelvetri et al., 2020; Evans and Liu, 2021; Stebbings et al., 2022; Sun et al., 2020; Wang et al., 2021). In addition, a number of cellular factors have been suggested to be involved in the viral entry of SARS-CoV-2, including non-muscle myosin heavy chain IIA (Myosin Heavy Chain 9, MYH9), heparan sulfate proteoglycans (HSPGs), Sialic acid, and integrins (Chen et al., 2021; Hu et al., 2021; Li et al., 2017). Integrins have been suggested to be involved in SARS-CoV-2 infection, transmission and pathology, and the conserved RGD motif (Arg-Gly-Asp, 403–405) in the receptor binding domain (RBD) of the spike glycoprotein of SARS-CoV-2 has been proposed to be an integrin binding site (Hussein et al., 2015; Kliche et al., 2021; Luan et al., 2020; Makowski et al., 2021).

Integrin (ITG) is a heterodimer transmembrane protein consisting of α subunit (containing 18 subtypes) and β subunit (containing 8 subtypes), and a Ca^2+^ or Mg^2+^ dependent cell adhesion factor that medicate the recognition of extracellular matrix and cell-surface ligands (Humphries, 2000). The I domain or β-Propeller domain of the α subunit and the βA domain of the β subunit of integrin could form the ligand binding domain (Campbell and Humphries, 2011; Hynes, 2002). In addition, integrins have been shown to be used as receptors or co-receptors for a number of viruses, such as human immunodeficiency virus 1 (HIV-1), foot-and-mouth virus, human cytomegalovirus, and adeno-associated virus type 2 (Arthos et al., 2008; Feire et al., 2004; Kotecha et al., 2017; Summerford et al., 1999; Wang et al., 2005). Computational analysis and clinical meta-analysis have identified the potential interactions between the RGD motif of SARS-CoV-2 and integrins (Aguirre et al., 2020; Jena et al., 2022; Sigrist et al., 2020; Wang et al., 2020). Moreover, experimental studies have provided evidence supporting the contributions of integrins on the viral binding (Amruta et al., 2021; Beddingfield et al., 2021; Huntington et al., 2022; Norris et al., 2023; Pham et al., 2021), For example, integrin α5β1 was reported to be a cellular receptor independent of ACE2 (Biering et al., 2022; Liu et al., 2022; Robles et al., 2022), whereas integrin αvβ3 was suggested to mediate a clathrin-dependent endocytosis without ACE2 (Bugatti et al., 2022; Nader et al., 2021; Simons et al., 2021). ACE2 expression level was reported to be positively correlated with increased expression level of integrin β3 in lung tissues with severe COVID-19 infection (Gheware et al., 2022). Integrin αvβ5 was found to be highly enriched in human intestines that may affect the expression of ACE2 and its binding to SARS-CoV-2, which may be associated with the low diarrhea rate in COVID-19 patients (Gao et al., 2021). While some studies reported that the α5, β1 and β3 subunits could exhibit binding function alone (Bristow et al., 2020; Kliche et al., 2021; Nader et al., 2021; Park et al., 2021), others demonstrated the necessity of activation of integrins (Dakal, 2021; Simons et al., 2021). However, the exact roles and underlying mechanisms of integrins in SARS-CoV-2 infection remain unclear and controversial, probably due to its great diversity and organ specificity.

Here, we examined the expression levels of various RGD-binding integrins in a panel of human cell lines and tested the susceptibility of these cell lines to SARS-CoV-2 using the pseudotyped viral system. We found that integrin αvβ1 was highly enriched in the susceptible cell lines. We performed additional studies and demonstrated that integrin αvβ1 significantly facilitated the ACE2-mediated SARS-CoV-2 entry in human Calu-3 and 293T cells as well as mouse NIH3T3 cells. This study may enhance our understanding of the pathogenesis of COVID-19 and offer potential therapeutic target for COVID-19.

## 2 Material and methods

### 2.1 Plasmids

Codon-optimized cDNA encoding the spike protein of SARS-CoV-2 (NCBI: NC_045512), SARS-CoV-2 RGD (403–405)→AAA variant, Gamma (NCBI: MZ477758), Delta (NCBI: MZ377115), Omicron (NCBI: OL672836) and VSV-G was synthesized and cloned into the eukaryotic cell expression vector pcDNA3.1(+) or pCMV3 (TSINGKE, China), respectively. The complete coding sequence (CDS) of the spike protein of SARS-CoV-2 variants were retrieved from the database of NCBI Virus (https://www.ncbi.nlm.nih.gov/labs/virus/). The lentiviral packaging plasmid psPAX2 was obtained from Huayueyang, China. The pLenti-GFP-luci lentiviral reporter plasmid that expresses GFP and luciferase was purchased from TSINGKE, China. The plasmids of the pLVX-puro, pLVX-puro-ACE2, pLVX-puro-ITGAV, and pLVX-puro-ITGB1 were also obtained from TSINGKE. All plasmid constructs used in this study were confirmed by DNA sequencing.

### 2.2 Cell lines and cell culture

The 293T (human embryonic kidney cells), SW620 (human colon cancer cells), IOSE-80 (human ovarian cancer cells), MDA-MB-231 (human breast cancer cells), HepG2 (human liver cancer cells), PANC-1 (human pancreatic cancer cells), HGC-27 (human gastric cancer cells, undifferentiated), BEAS-2B (human normal lung epithelial cells), Calu-3 (human lung adenocarcinoma cells) and NIH3T3 (mouse embryonic fibroblast cells) were obtained from BeNa culture collection (BNCC, China). The cells were maintained in their respective medium (SIGMA, Germany) with 10% fetal bovine serum (FBS, Biological Industries, America) and 1% Penicillin-Streptomycin (PS) (100 × Solution, Hyclone, America). Cell cultures were maintained in a humidified incubator at 37 °C in 5% CO_2_ in the indicated media and passaged every 3–4 days.

The stable cell lines of 239T cells with empty vector (239T/vector), 239T cells stably expressing recombinant human ACE2 (293T/hACE2), NIH3T3 cells with empty vector (NIH3T3/vector), and NIH3T3 cells stably expressing recombinant human ACE2 (NIH3T3/hACE2) were established using the lentivirus system, respectively (Benskey and Manfredsson, 2016). All the stable cell lines were cultured under the selection with puromycin.

### 2.3 Production of pseudotyped virions

The pseudotyped virions were produced by co-transfection of 293T cells with psPAX2, pLenti-GFP-luci, and plasmids encoding coronavirus spike or VSV-G using Lipo8000 (Beyotime, China) (Benskey and Manfredsson, 2016). The supernatants were harvested at 72 h or 96 h post transfection, passed through 0.45 μm filter, and centrifuged at 3500 rpm for 30 min to remove the cell debris. Then the supernatants were stored at 4  °C overnight after the addition of PEG-6000 at a final concentration of 8.5% and NaCl at a final concentration of 0.3 M. The mixtures were centrifuged at 3500 rpm for 30 min. The viral pellets were resuspended in PBS, aliquoted, and stored at −80 °C for subsequent studies. The titer of the pseudotyped virions was determined by p24 ELISA kit (Biodragon, China).

### 2.4 Viral entry assay

For viral entry assay, 2 × 10^4^ cells per well were seeded in a 96-well plate for one day, cells were inoculated with 10μl media containing the pseudotyped virions (Multiplicity of Infection, MOI = 2.5) overnight. Then the medium was removed with addition of fresh medium, and cells were incubated for another 48 h and analyzed for luciferase activity using the ONE-Glo™ Luciferase Assay System (Promega, USA) by a GloMax luminometer (Promega, USA). To further investigate the contributions of the receptor usage for viral entry, the cells were incubated with 1 μg/ml or 10 μg/ml of different antibody (Ab) that targets the respective receptor (Supplementary Table 2) at 37 °C for 1 h before inoculation with the pseudotyped virions.

### 2.5 mRNA expression analysis

Reverse transcription quantitative real-time PCR (RT-qPCR) was performed to detect the mRNA expression level of the target gene according to the manufacturer's instructions. In brief, total RNA was first extracted using the TRIzol Reagent (SIGMA, Germany), which was then converted to complementary DNA (cDNA) using a FastKing RT Kit (with gDNase) (TIANGEN, China). The qPCR was performed using 2X ChamQ Universal SYBR qPCR Master Mix Kit (Vazyme, China) with the QuantStudio DX Real Time System (Thermo Fisher Scientific, USA). All primers used in this study were listed in Supplementary Table 1.

### 2.6 Analysis of protein expression

Western blotting (WB) was used to detect the protein expression level. Briefly, cell protein lysates were prepared in RIPA buffer (KeyGEN Biotech, China) with 1 × protease inhibitor cocktail (MedChemExpress, USA). After measuring the protein concentration using a BCA kit (Thermo Fisher Scientific, USA), 5 × loading buffer (Beyotime, China) was added. Then these samples were boiled for 10 min and separated in a 10% SDS-PAGE gel and transferred to PVDF/nitrocellulose filter membranes (Immobilon^@^-P Membrane, IPVH00010). After blocked by 5% milk at 4 °C for 2 h, the membranes were blotted with primary antibodies overnight at 4 °C. Following 0.1% Tween-20 (TBST) washing, the membranes were incubated with their respective horseradish peroxidase (HRP) conjugated secondary antibodies for 1 h at room temperature. After washing, the signal was detected with SuperSignalTM West Femto Maximum Sensitivity Substrate (Thermo Fisher Scientific, USA)/chemiluminescence (ECL) kit (Pierce, USA) and visualized with Chemiluminescent Reagent (Bio-Rad).

For immunoprecipitation (IP) and co-IP assays, 293T cells were seeded into 60 mm culture dish plate and transfected with plasmids of RBD-flag, RBD-RGD (403–405)→AAA-flag, ITGAV, and ITGB1 respectively when cells reached 70–80% confluence. The cell lysate was prepared at 48 h post transfection and the supernatants were collected by centrifugation at 4 °C and quantified by the BCA assay. Briefly, mouse anti-flag antibody and 40 μL protein A/G agarose beads (Beyotime, China) were added into 600 μL (1 μg/μL) supernatants and then incubated at 4 °C overnight. The beads were washed completely, and the co-IP product was obtained with 60 μL of 2 × SDS-PAGE loading buffer. After incubation for five minutes at 100 °C, 15 μL of the co-IP products was used for western blotting analysis using anti-ITGAV and anti-ITGB1 antibodies.

The Flow Cytometry was performed to detect the expression level of cell surface proteins. Briefly, cells were incubated with primary antibodies at 37 °C for 1 h, followed by secondary antibodies for 1 h. Then cells were washed twice with PBS containing 2% FBS and 2 mM EDTA (Sangon Biotech, China), and analyzed by using a BD Accuri C6 flow cytometer and software (BD Biosciences, San Jose, CA, USA). All antibodies were listed in Supplementary Table 2.

### 2.7 Bioinformatics analysis

GEPIA2 (http://gepia2.cancer-pku.cn/#index) database was used to visualize the gene expression. The SARS-CoV-2 spike glycoprotein structure QHD43416 from I-TASSER (https://www.zhanggroup.org/COVID-19/) based on experimental structure (PDB ID: 6VYB, 6VXX, 6LXT) was used for the modeling of SARS-CoV-2 RGD (403–405)→AAA variant spike (Roy et al., 2010). The structure of integrin A5B1 (PDB ID:3VI4) and integrin AV (PDB ID:6UJC) were used as the template for building homology model of integrin AVB1. Modeling was performed via the website Swiss Model (https://www.swissmodel) with the default parameters (Waterhouse et al., 2018). Computational modeling of protein–protein complexes docking predictions were performed using HADDOCK server (https://bianca.science.uu.nl/haddock2.4/) with the default parameters. The most reliable docking model was selected by the Z-score according to HADDOCK (de Vries et al., 2010). The structure alignment have been performed and displayed by PyMOL (Lill and Danielson, 2011). We used the Molecular Mechanics-Poisson Boltzman Surface Area (MM-PBSA) method to calculate the binding free energy for molecular dynamics (MD) analysis. The complex structures were subjected to classical molecular dynamics for 50 ns for the stabilization. The MM-PBSA free binding energy between spike proteins and hACE2 (PDB ID:6M1D) was calculated using the prime module of Schrodinger software (Genheden and Ryde, 2015).

### 2.8 Statistical analysis

All experiments were done in triplicates, and repeated at least twice or more. The data were analyzed using Prism 5.0 software (GraphPad, USA). Data are expressed as the mean ± standard error of the mean (SEM). Statistical significance between two groups was determined by unpaired two-tailed Student's *t*-test and multiple group comparisons were performed using one-way analysis of variance (ANOVA). Differences were considered to be significant when *P*<0.05 (indicated with one asterisk (*)), *P*<0.01 (indicated with two asterisks (**)), *P*<0.001 (indicated with three asterisks (***)).

## 3 Results

### 3.1 High expression of integrin αvβ1 in SARS-CoV-2 susceptible cell lines

**Fig. 1:**
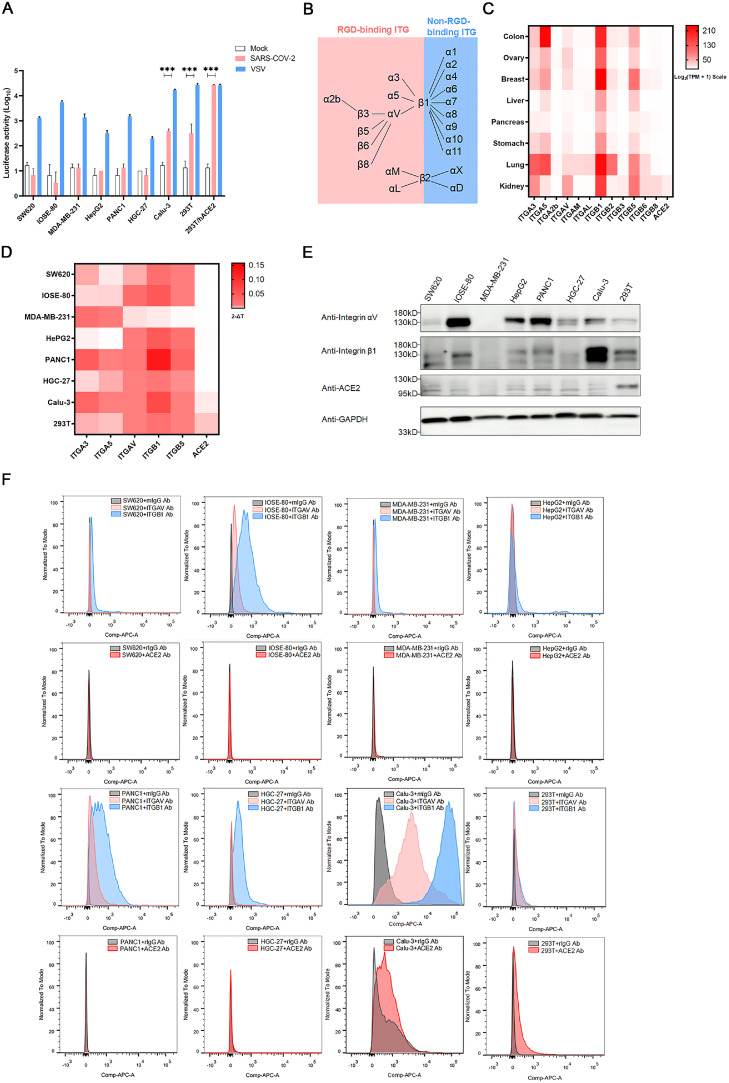
The high expression of integrin αvβ1 in the SARS-CoV-2 susceptible cell lines. A. Entry of SARS-CoV-2 S pseudotyped virions into human cell lines SW620, IOSE-80, MDA-MB-231, HepG2, PANC-1, HGC-27, BEAS-2B, Calu-3, 293T, and 293T/hACE2). Cells were infected with SARS-CoV-2 S-pseudotyped (red) and VSV-pseudotyped viruses, respectively, or mock infection (white). At 72 h post infection, viral entry efficiency was measured by luciferase activity assay. Significant difference from mock were determined by two-tailed unpaired t-test. ***P < 0.001. Error bars indicate SD (*n* = 3). B. A schematic diagram showing the RGD- and non-RGD binding integrins. C. Heatmap of the mRNA expression profile of RGD-binding integrins in different human organs obtained from the GEPIA2 database. The mRNA expression was exhibited by Log2(TPM+1) scale, and TPM denotes Transcripts Per Million. D. Heatmap of the mRNA expression profile of integrin ITGA3, A5, AV, B1, B5, and ACE2 in indicated cell lines detected by RT-qPCR. The mRNA expression levels were normalized to the level of GAPDH. E. The protein expression level of integrin ITGAV, B1, and ACE2 in indicated cell lines was analyzed by western blot with anti-integrin av, β1, and ACE2 antibodies, respectively. F. The expression levels of ITGAV and ITGB1 on the cell membrane were analyzed by flow cytometry. Histograms indicate the expression of integrins. Red: anti-ACE2 Ab, pink: anti-integrin av Ab, blue: anti-integrin β1 Ab, gray: isotype-control Ab.

To identify the potential integrins involved in SARS-CoV-2 entry, we first determined the susceptibility of various cell lines to SARS-CoV-2 infection using a pseudotyped lentiviral system (Fig. 1A). The pseudotyped-VSV served as a positive control for viral entry. Considering the multi-organ infection in COVID-19 cases (Puelles et al., 2020), a panel of human cell lines derived from different organs were used for pseudotyped-SARS-CoV-2 infection assay, including SW620 (human colon cancer cells), IOSE-80 (human ovarian cancer cells), MDA-MB-231 (human breast cancer cells), HepG2 (human liver cancer cells), PANC-1 (human pancreatic cancer cells), HGC-27 (human gastric cancer cells), Calu-3 (human lung adenocarcinoma cells), 293T (human embryonic kidney cells, 293T). The 293T/hACE2 cells that overexpressed the major receptor ACE2 were used as a positive control (Supplementary Fig. 1A, 1B, 1C). The results showed that 293T/hACE2 (*P* = 9.75E-04), Calu-3 (*P* = 3.01E-04), and 293T cells (*P* = 2.22E-05) were permissive to SARS-CoV-2 pseudotyped virus infection in vitro (Fig. 1A).

It has been proposed that an integrin recognition motif RGD (403–405) in the SARS-CoV-2 spike protein may mediate the interaction between SARS-CoV-2 RGD and the RGD-dependent binding integrins, such as αvβ1, αvβ3, αvβ5, α5β1, αvβ6, αvβ8, and αIIbβ3 (Fig. 1B) (Yan et al., 2020). Thus, we evaluated the mRNA expression level of RGD-dependent binding integrins in several human organs via GEPIA2 database. As shown in Fig. 1C, ITGB1, B5, A3, AV and A5 were highly expressed in various organs. We then investigated the mRNA expression level of ITGB1, B5, A3, AV and A5 in cell lines used in the infection assay by qPCR. As expected, ACE2 was only detected in susceptible cell lines Calu-3 and 293T, whereas ITGA3, ITGA5, ITGAV, ITGB1 and ITGB5 were differentially expressed in various cell lines (Fig. 1D). We further examined the total protein expression level and the cell surface protein expression level of integrin αvβ1 by WB (Fig. 1E) and flow cytometry (Fig. 1F), respectively, and the results were consistent with the qPCR results. Of note, although Calu-3 had a relatively lower expression level of ACE2 than 293T cells (*P* = 5.07E-03), there was no significant difference in susceptibility between these two cell lines (*P* = 9.16E-02). However, integrin β1 expression level in Calu-3 was higher than 293T (*P* = 1.17E-04), indicating the potential promotion effect of integrin β1 on the ACE-2 mediated cellular entry of SARS-CoV-2. Taken together, our data suggest that integrin αvβ1 may promote the SARS-CoV-2 infection.

### 3.2 Interaction between the RGD motif on SARS-CoV-2 and integrin αvβ1

**Fig. 2:**
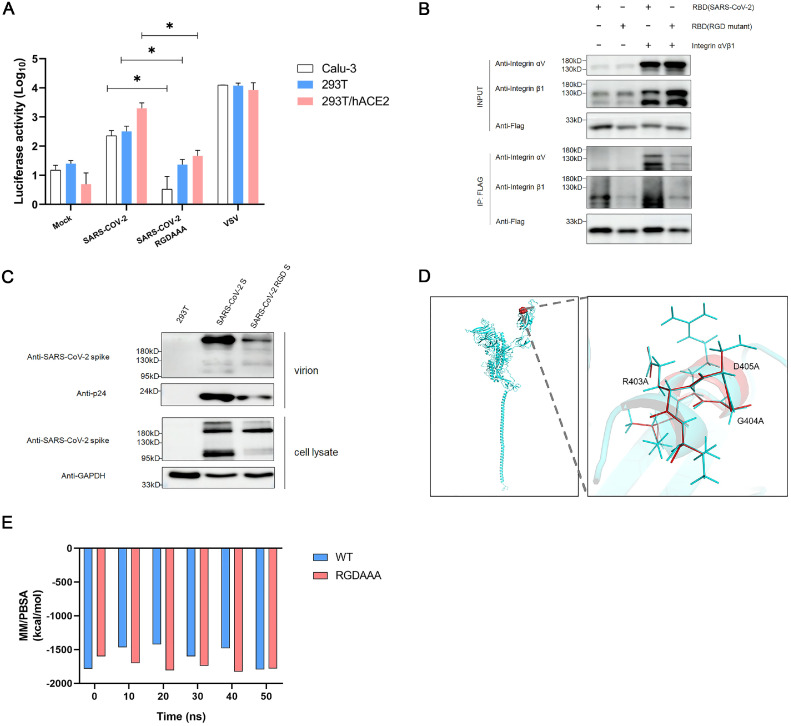
The interaction between RGD motif on SARS-CoV-2 and integrin αvβ1. A. Three susceptible cell lines (Calu-3, 293T and 293T/hACE2) were infected with SARS-CoV-2 wild type (WT) and the RGD (403–405)→AAA mutant S pseudotyped viruses, respectively. The luciferase activity (LUC) was measured at 72 h post infection. The VSV-pseudotyped viruses were used as a positive control whereas the mock infection served as a negative control. Significant difference from wild-type were determined by two-tailed unpaired t-test. ***P < 0.001. Error bars indicate SD (*n* = 3). B. Detection of interaction between the RGD motif on RBD of SARS-CoV-2 spike and integrin αvβ1 by immunoprecipitation (IP). 293T cells were transfected with integrin av, integrin β1, Flag-tagged WT RBD, and Flag-tagged RGD (403–405)→AAA mutant RBD, respectively. The cell lysates for IP were prepared at 48 h post transfection. RGD (403–405)→AAA mutant is defective in binding to integrin αvβ1 compared to its wild type counterpart. C. Detection of WT S or the RGD(403–405)→AAA mutant S proteins in the pseudotyped virions by western blot with antibodies against SARS-CoV-2 spike protein. D. Structural alignment of WT SARS-CoV-2 S and the RGD (403–405)→AAA variant. (I) Mutant region on spike protein. (II) Enlarged view of structural alignment of mutant region (blue: wild type; red: RGD (403–405)→AAA variant). E. MM/PBSA binding free energy of SARS-CoV-2 RBD-hACE2 complex and RGD variant RBD-hACE2 complex for 50 ns.

To detect the interaction between the RGD motif in SARS-CoV-2 and integrin αvβ1, we generated the pseudotyped viruses bearing the S protein with the wild type (WT) RGD and the RGD (403–405)→AAA variant, respectively, and examined the infectivity of these two pseudotyped viruses in Calu-3, 293T and 293T/hACE2 cells (Fig. 2A). The viral entry efficiency of RGD (403–405)→AAA variant was significantly reduced in all susceptible cell lines (Calu-3, *P* = 2.87E-02; 293T, *P* = 3.61E-02; 293T/hACE2, *P* = 3.42E-02). In addition, we performed Co-IP experiments using FLAG-tagged SARS-CoV-2 RBD, FLAG-tagged SARS-CoV-2 RGD (403–405)→AAA variant RBD, ITGAV, and ITGB1. Fig. 2B showed that SARS-CoV-2 RBD exhibited potent binding to integrin αvβ1, whereas the association of RGD (403–405)→AAA variant RBD with integrin αvβ1 was dramatically reduced.

To understand the possible influence of RGD (403–405)→AAA variant on the incorporation of S protein into the pseudotyped virions, we examined the levels of the S and p24 proteins in the virions and the S proteins in the cell lysate by western blot. The results revealed that the full-length S proteins (∼180 kDa) were detected in both the WT RGD and RGD (403–405)→AAA pseudotyped virons, but the level of the mutant RGD was markedly reduced compared with the WT one (Fig. 2C). Based on the observation that the level of p24 protein in the mutant RGD was also significantly decreased, we reasoned that the variation in the virions’ input may contribute to the “incorporation difference” between the WT S and RGD (403–405)→AAA variant S. In addition, the cleaved S protein product (∼95 kDa) was also decreased significantly (Fig. 2C), suggesting that the RGD (403–405)→AAA mutation may affect the cleavage state of the S protein. Furthermore, we also analyzed the S protein structure and simulated WT RBD-hACE2 complex and the RGD (403–405)→AAA variant RBD-hACE2 complex for 50 ns to investigate the dynamics, finding that the RGD (403–405)→AAA mutation didn't cause significant change of the secondary structure and binding energy (Fig. 2D, 2E). Over the 50 ns of simulations, the total binding energy in RGD (403–405)→AAA RBD-hACE2 complex and WT RBD-hACE2 complex were −1780.209 kcal/mol and −1794.151 kcal/mol, respectively, which were relatively stable and showed no significant difference (Fig. 2E). In summary, the decrease in infectivity of RGD (403–405)→AAA variant was not related to ACE2, but was more likely due to the contribution of integrin αvβ1. These results suggest that a direct interaction between integrin αvβ1 and SARS-CoV-2 RGD (403–405) motif may affect the infectivity of SARS-CoV-2.

### 3.3 Anti-αvβ1 integrin antibodies inhibited viral entry of SARS-CoV-2 into cells

**Fig. 3:**
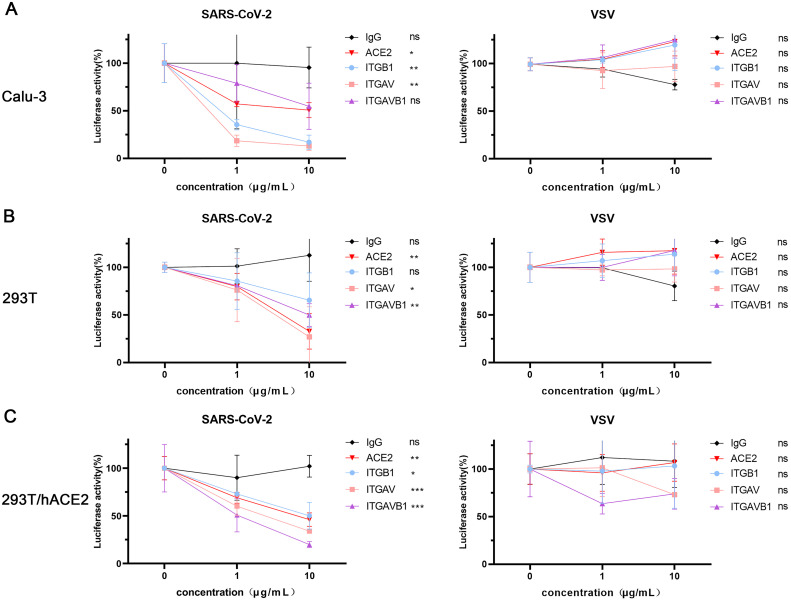
Anti-αvβ1 integrin antibodies inhibited the SARS-CoV-2 entry into the susceptible cell lines. A-C. Inhibition of SARS-CoV-2 S pseudotyped virus entry by anti-αvβ1 integrin antibodies in three susceptible cell lines, Calu-3 (A), 293T (B), and 293T/hACE2 (C). Cells were infected with SARS-CoV-2 S-pseudotyped (red) and VSV-pseudotyped viruses in the presence or absence of respective antibodies at various concentrations (0, 1, 10 μg/mL), respectively. Anti-ACE2 antibody was used as a positive control whereas the isotype-matched IgG served as a negative control. The viral entry efficiency was measured by luciferase activity assay at 72 h post infection. Significant difference between the untreated groups and the 10 μg/mL antibody treated groups were determined by two-tailed unpaired t-test. ns: not significant, *p* > 0.05; *P < 0.05; **P < 0.01; ***P < 0.001. Error bars indicate SD (*n* = 3).

To examine the role of αvβ1 integrin in the SARS-CoV-2 entry into host cells, we infected three susceptible cell lines (Calu-3, 293T and 293T/hACE2) with the pseudotyped viruses in the presence and absence of anti-αvβ1 integrin antibodies, anti-ACE2 antibody (a positive control), and the isotype control antibody (a negative control), respectively. In Calu-3 cells (Fig. 3A), anti-ACE2 (*P* = 1.78E-02), anti-αv (*P* = 1.99E-03) and anti-β1 (*P* = 2.74E-03) antibodies all exhibited significant blocking activities. Surprisingly, the anti-integrin antibodies had more potent inhibitory activities than the anti-ACE2 antibody. Approximately 85% reduction of the luciferase activities in the infected cell with anti-integrin antibodies (10μg/ml) whereas about 50% decrease in the cells treated with anti-ACE2 antibody (10μg/ml). However, the combination of anti-αv and anti-β1 integrin antibodies had no significant effect on the viral entry (*P* = 6.92E-02). In 293T cells (Fig. 3B), the anti-ACE2 (*P* = 3.93E-03), anti-αv integrin (*P* = 1.69E-02), and the combination of anti-αv and anti-β1 integrin antibodies (*P* = 2.73E-03) significantly inhibited the viral entry. However, the anti-β1 integrin antibody (*P* = 1.11E-01) did not show significant effect. In 293T/hACE2 cells (Fig. 3C), the anti-ACE2 (*P* = 2.16E-03), anti-αv (*P* = 5.73E-04), anti-β1 (*P* = 3.84E-02), and the combination of anti-αv and anti-β1 integrin antibodies (*P* = 7.77E-05) all significantly inhibited the viral entry. Of note, all the antibodies showed inhibitory effect in the respective cell lines acted in a dose dependent manner. Moreover, all the antibodies mentioned above had no significant inhibitory effect on the viral entry of the VSVG-pseudotyped viruses. Our results suggest that the anti-αvβ1 integrins may play an important role in the viral entry of SARS-CoV-2 into the susceptible cell lines.

### 3.4 Integrin αvβ1 facilitates ACE2-mediated viral entry of SARS-CoV-2

**Fig. 4:**
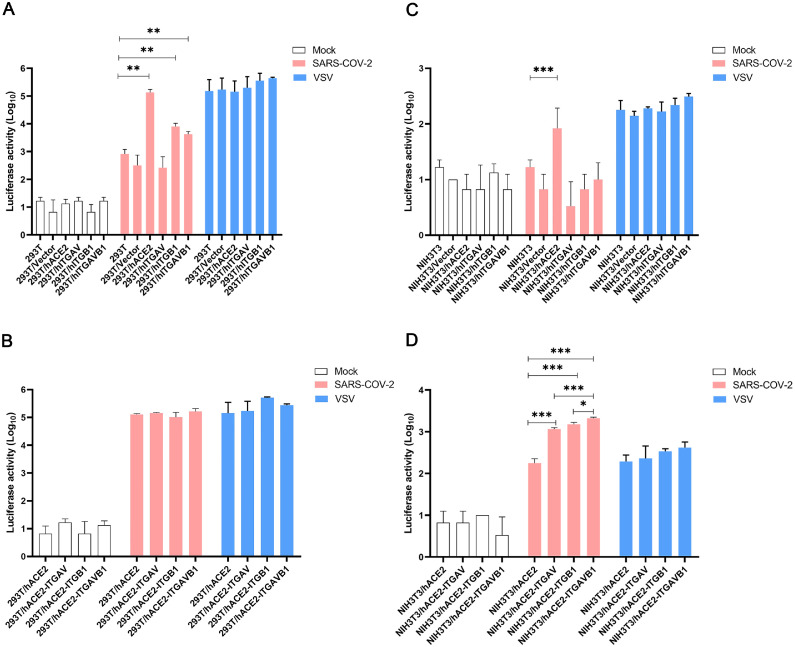
Integrin αvβ1 facilitates SARS-CoV-2 virus entry into cells A. The 293T cells stably expressing human ACE2, integrin αv, integrin β1, or integrin αvβ1 were infected with mock (white), SARS-CoV-2 S-pseudotyped (red), and VSV-pseudotyped viruses (blue), respectively. B. The 293T cells stably expressing human ACE2 (293T/hACE2) were transiently transfected with integrin αv, integrin β1, or integrin αvβ1, followed by infection of mock (white), SARS-CoV-2 S-pseudotyped viruses (red), and VSV-pseudotyped viruses (blue), respectively. C. The NIH3T3 cells stably expressing human ACE2, integrin αv, integrin β1, or integrin αvβ1 were infected with mock (white), SARS-CoV-2 S-pseudotyped (red), and VSV-pseudotyped viruses (blue), respectively. D. The NIH3T3 cells stably expressing human ACE2 (NIH3T3/hACE2) were transiently transfected with integrin αv, integrin β1, or integrin αvβ1, followed by infection of mock (white), SARS-CoV-2 S-pseudotyped viruses (red), and VSV-pseudotyped viruses (blue), respectively. The viral entry efficiency was measured by luciferase activity assay at 72 h post infection. Significant difference between the groups were determined by two-tailed unpaired t-test. ns: not significant, *p* > 0.05; *P < 0.05; **P < 0.01; ***P < 0.001. Error bars indicate SD (*n* = 3).

**Fig. 5:**
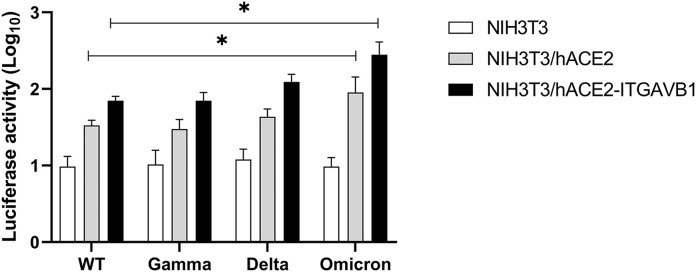
The effect of integrin αvβ1 on the viral entry of different SARS-CoV-2 variants. NIH3T3, NIH3T3/hACE2 and NIH3T3/hACE2-ITGAVB1 cells were infected with different pseudotyped SARS-CoV-2 variants (wild-type, Gamma, Delta and Omicron), respectively. The viral entry efficiency was measured by luciferase activity assay at 72 h post infection. Significant difference between the groups were determined by two-tailed unpaired t-test. *P < 0.05; Error bars indicate SD (*n* = 3).

**Fig. 6:**
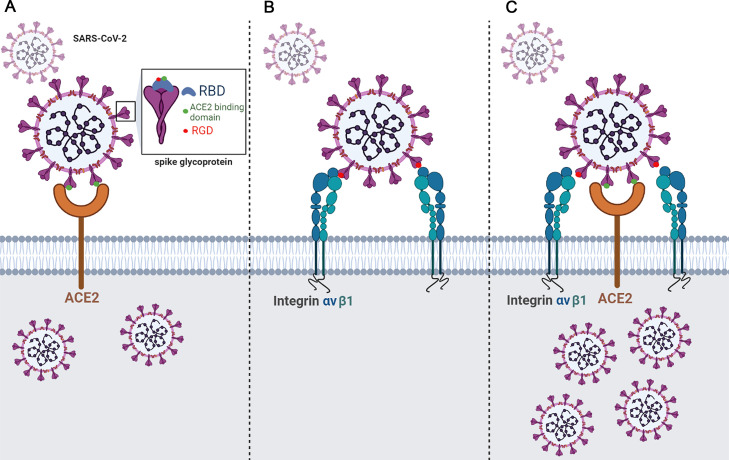
A proposed model illustrating the action and mechanism of integrin αvβ1 that facilitates ACE2-mediated viral entry of SARS-CoV-2 A. ACE2 functions as an independent receptor for SARS-CoV-2 entry into the host cells; B. Integrin αvβ1 is unable to serve as an independent receptor for SARS-CoV-2 entry into the host cells; C. Integrin αvβ1 may significantly enhance the ACE2-mediated viral entry of SARS-CoV-2.

To determine whether integrin αvβ1 can function as an independent receptor or as a co-receptor for SARS-CoV-2 entry into cells, we infected 293T, 293T/empty vector, 293T/hACE2, 293T/integrin αv, 293T/integrin β1, and 293T/integrin αvβ1, respectively, with the pseudotyped viruses. In 293T cells, transient expression of human ACE2 (*P* = 3.94E-03), integrin β1 (*P* = 7.88E-03) and integrin αvβ1 (*P* = 7.35E-03) significantly increased the viral entry, whereas there was no significant change in the 293T cells expressing integrin αv (Fig. 4A). However, in the 293T cells stably expressing human ACE2 (i.e. 293T/hACE2 cell line), we did not observe significant effect of integrin αv, integrin β1 and integrin αvβ1 on the ACE2-mediated viral entry (Fig. 4B). In addition, we conducted similar viral entry studies on mouse NIH3T3 cells that transiently or stably expressing human ACE2, integrin αv, integrin β1, and integrin αvβ1, respectively (Fig. 4C and Fig. 4D). Of note, NIH3T3 cells are mouse embryonic fibroblast cells that do not express human ACE2 and integrin αvβ1 (Supplementary Figure 2A, 2B). While the NIH3T3 cells expressing human ACE2 exhibited significant viral entry (*P* = 4.06E-03), the NIH3T3 cells expressing integrin αv, integrin β1 or integrin αvβ1 had no significant effect on the viral entry, suggesting that integrin αv, integrin β1 or integrin αvβ1 could not function as an independent receptor for the viral entry of SARS-CoV-2 (Fig. 4C). Additional studies using the NIH3T3 cells stably expressing human ACE2 demonstrated that transient expression of integrin αv, integrin β1 or integrin αvβ1 may significantly enhance the ACE2-medicated viral entry of SARS-CoV-2 (Fig. 4D). Furthermore, we investigated the contribution of integrin αvβ1 to the ACE2-mediated viral entry of the variants of concerns (VOCs) and found that the Omicron showed a significant increase in entry into the NIH3T3/hACE2 (*P* = 3.58E-02) and NIH3T3/hACE2-ITGAVB1 (*P* = 1.61E-02) cells compared with the WT one, respectively (Fig. 5). We also performed Omicron entry assay in 293T cells (Supplementary Figure 3) and found that Omicron showed a significant increase (*P* = 8.60E-03) in entry into 293T/hACE2-ITGAVB1 compared with 293T/hACE2, which was consistent with the findings in NIH3T3/ hACE2-ITGAVB1 cells (Fig. 5). These results suggest that the effect of integrin αvβ1 on the Omicron entry is not cell line dependent. Of note, the Omicron strain used in this study is Omicron BA.1, which contains the RGD (403–405) motif. Taken together, our data indicated that ACE2 is the major cellular receptor of SARS-CoV-2 and its binding triggers the entry of the viral genome into the host cell, whereas αvβ1 integrin is more of an adhesion/attachment receptor to facilitate its binding to ACE2 (Fig. 6).

## 4 Discussion

A number of studies have demonstrated that integrins on the cell-surface may contribute to the high affinity of viral binding of the SARS-CoV-2, but the exact role of integrins in ACE2-mediated SARS-CoV-2 entry appears controversial, varying from the independent cellular receptor competing with ACE2 to the facilitator synergizing with ACE2 (Caccuri et al., 2021; Gao et al., 2021; Luan et al., 2020; Sigrist et al., 2020). In this study, we first examined the expression level of integrins containing the RGD-motif in a panel of human cell lines and tested the susceptibility of these cell lines to SARS-CoV-2 infection. We subsequently identified the integrin αvβ1 that were highly enriched in several susceptible cell lines.

Integrin α3β1, α5β1, and αvβ5 were also enriched in Calu-3 cells, which is consistent with the findings of high abundance in lung tissues based on the database analysis (unpublished data). In addition, α5β1 and αvβ5 integrins have been shown to be involved in SARS-CoV-2 infection in the respiratory system (Liu et al., 2022; Nader et al., 2021), suggesting that multiple integrins instead of one type may act jointly on SARS-CoV-2 infection. Of note, we also detected the expression of ACE2 and αvβ1 integrin in some non-permissive cell lines (e.g. IOSE-80 and PANC-1) by Western blot and qPCR, but they were undetectable on the cell surface by flow cytometry analysis, which may help explain their insusceptibility to SARS-CoV-2 infection. Moreover, the differential expression of integrin αvβ1 between Calu-3 and 293T cells probably contributed to the difference in the inhibitory activities by the respective antibodies. While the anti-β1 integrin antibody significantly reduced the SARS-CoV-2 infectivity in Calu-3, it did not show similar effect on 293T cells. The integrin β1 in 293T was obviously lower than in Calu-3. In addition, there was a disparity of integrin β1expression levels in the total protein and membrane protein testing results between Calu-3 and 293T. Although the combination of the anti-αv and anti-β1 integrin antibodies did not significantly block the infection in Calu-3 cells, the downtrend was apparent. This may be also related to the high expression of multiple integrins in Calu-3 cells. Furthermore, the susceptibility of different cell types to SARS-CoV-2 infection may be modulated by the conformation of relevant integrins and the impact of the tissue microenvironment on the spike protein conformation (Makowski et al., 2021).

The RGD motif in the spike protein of SARS-CoV-2 has been suggested to be essential element for virus internalization (Yan et al., 2020). Here, to investigate interaction between the RGD motif and integrin αvβ1, we generated the pseudotyped-SARS-CoV-2 RGD (403–405)→AAA variant. The selection of alanine (Ala) amino acid mutations in the RGD region is because that Ala (A) is a chiral amino acid with the shortest side chain (R group is CH_3_), and the mutation of an amino acid residue to Ala-has been widely used to investigate the function of the mutant protein in many studies (Yang et al., 2005). Interestingly, the RGD (403–405)→AAA mutation not only markedly affected the viral entry, but also the cleavage of the spike protein of SASR-CoV-2. Previous studies reported that Foot-and-mouth disease virus (FMDV) was cleaved in situ by trypsin at the RGD motif (Gullberg et al., 2013; Hernández et al., 1996), suggesting that other cleaving methods may exist at the RGD motif (403–405) near the acknowledged S cleavage site (672, 94–699, 709), which warrants further studies. Although the dynamic simulation analysis showed that the RGD (403–405)→AAA mutation didn't cause significant change of the binding energy of RBD-ACE2 complex, we should consider the possible limitations of the simulation software that did not take into account the contributions of other receptor binding factors for SARS-CoV-2.

There are some discrepancies between 293T and NIH3T3 cells regarding the contributions of integrin αvβ1 to the ACE2-meddiated viral entry. For instance, there was no significant difference in the viral entry efficiency between 293T/hACE2 and 293T/hACE2+ integrin αvβ1 (Fig. 4B), whereas the NIH3T3/hACE2+integrin αvβ1 was significantly higher than the NIH3T3/hACE2 (Fig. 4D). It's possible that 293T/hACE2 cells have already had certain level of endogenous αvβ1 integrin and the transient expression did not help. On the other hand, the mouse NIH3T3 cells do not express endogenous human αvβ1 integrin, and therefore the transient expression resulted in a significant increase in the ACE2-mediated viral entry.

Our data suggested that integrin αvβ1 could not function as an independent receptor for the cellular entry of SARS-CoV-2, but it can serve as a facilitator for the ACE2-mediated viral entry through the interaction with the RGD motif in the spike protein of SARS-CoV-2. Our finding that the Omicron exhibited a significant increase in the ACE2-mediated viral entry is consistent with results from the inhibition study of the integrin signaling by Huntington KE et al. (Huntington et al., 2022). In addition, Makowski L et al. proposed that the emergence of receptor-binding domain mutations that increase infectivity may also enhance the access of the RGD motif for integrin binding (Makowski et al., 2021). Since the virus transmission efficiency is directly correlated to the affinity of the virus to its host cell receptor, our study may help explain the high infectivity and widespread extra-pulmonary impacts of Omicron to some extent. Of note, in addition to the Omicron BA.1 used in this study, other Omicrons, such as BA.2, BA.5, XBB.1.5, lost the RGD (403–405) motif and possessed RGN (403–405). It would be interesting to examine how RGN (403–405) motif may affect the ACE-2 mediated viral entry of the Omicrons in future studies. Another limitation of this study is that we only used the established cell lines infected by the pseudotyped viruses, which may not represent the real status of the viral entry events. Therefore, additional validation studies are warranted by using human primary cells or patient-derived organoid (PDO) infected by the wild type or mutant SARS-CoV-2.

To our best knowledge, this is the first study reporting that integrin αvβ1 may significantly facilitate ACE2-mediated entry of SARS-CoV-2. Our findings may not only help to dissect the cellular factors involved in the viral entry of SARS-CoV-2, but may also enhance our understanding of the pathogenesis of COVID-19.

## CRediT authorship contribution statement

**Zeqiong Cai:** Methodology, Writing – original draft. **Han Bai:** Methodology, Writing – original draft. **Doudou Ren:** Methodology, Writing – original draft. **Biyun Xue:** Methodology. **Yijia Liu:** Methodology. **Tian Gong:** Methodology. **Xuan Zhang:** Methodology. **Peng Zhang:** Methodology. **Junsheng Zhu:** Methodology. **Binyin Shi:** Supervision, Funding acquisition. **Chengsheng Zhang:** Conceptualization, Methodology, Resources, Writing – original draft, Writing – review & editing, Supervision, Funding acquisition, Project administration.

## Declaration of Competing Interest

The authors declare that they have no known competing financial interests or personal relationships that could have appeared to influence the work reported in this paper.

## Data Availability

Data will be made available on request.

## Appendix Supplementary materials

Image, application 1
