# Supplementary material for: Integrin αvβ1 facilitates ACE2-mediated entry of SARS-CoV-2
Source: Virus Res. 2023 Nov 2;339:199251. doi: 10.1016/j.virusres.2023.199251 (PMC10651773; doi:10.1016/j.virusres.2023.199251)
Supplement: Supplementary file 1 [file mmc1.docx]

**Supplementary Material**


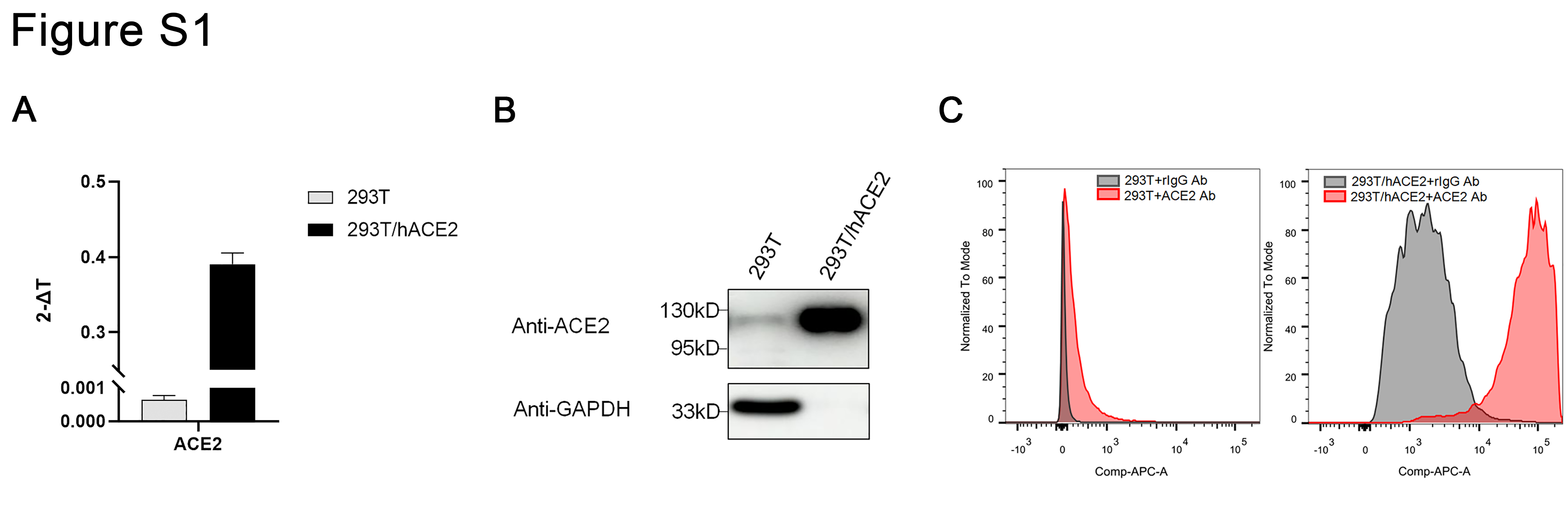


**Supplementary Figure 1 (SF1). Detection of ACE2 expression in 293T and 293T/hACE2 cells.**

1. The ACE2 expression levels in 293T and 293T/hACE2 were detected by qPCR;
2. The ACE2 expression levels in 293T and 293T/hACE2 (1/50) were detected by western blot;
3. The ACE2 expression levels in 293T and 293T/hACE2 were detected flow cytometry.

**
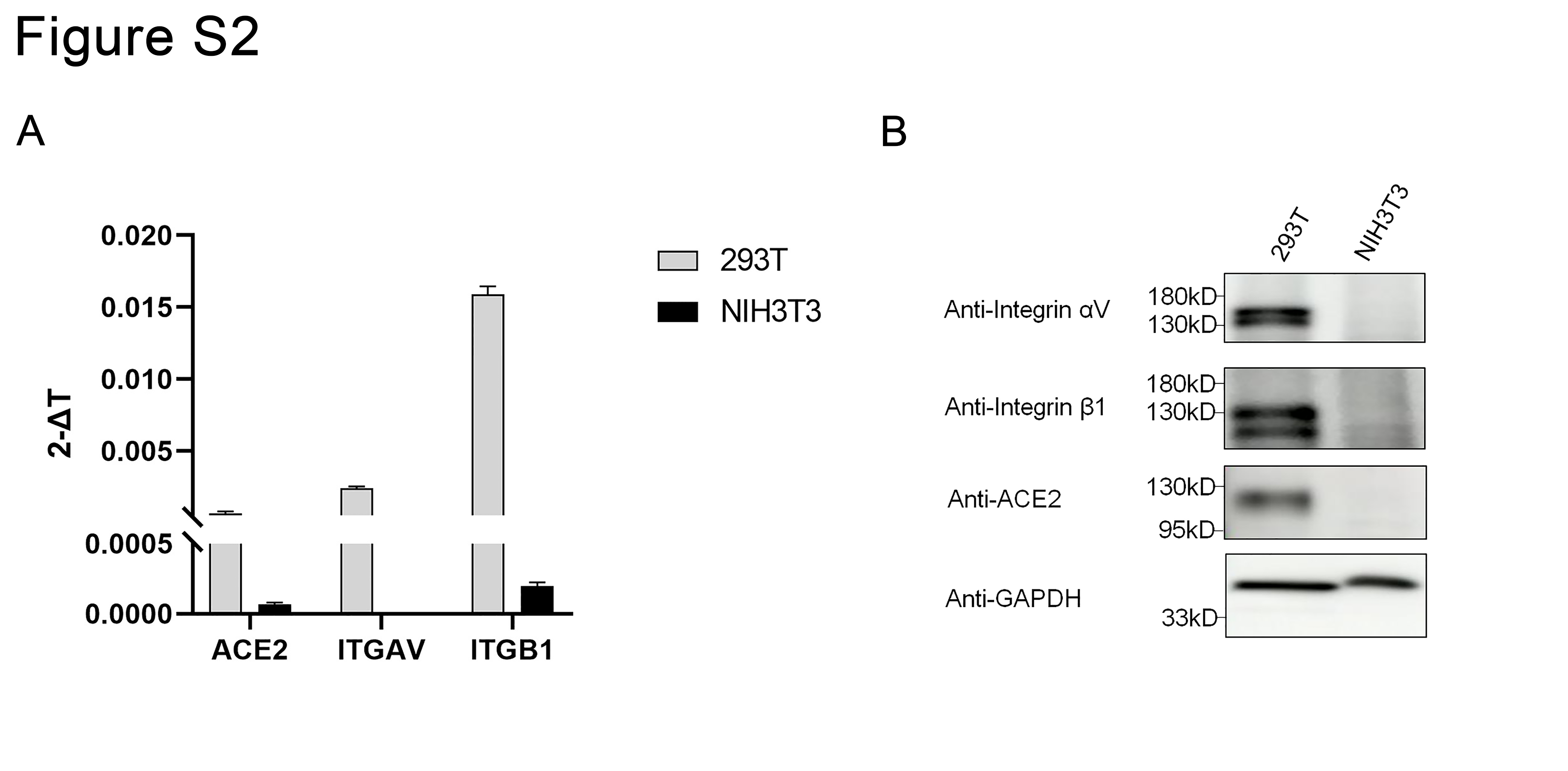
**

**Supplementary Figure 2 (SF2). Detection of ACE2, integrin αv, and integrin β1 in 293T and NIH3T3 cells.**

1. The expression levels of ACE2, integrin αv and integrin β1 in 293T and NIH3T3 cells were detected by qPCR;
2. The expression levels of ACE2, integrin αv and integrin β1 in 293T and NIH3T3 cells were detected by western blot.

**
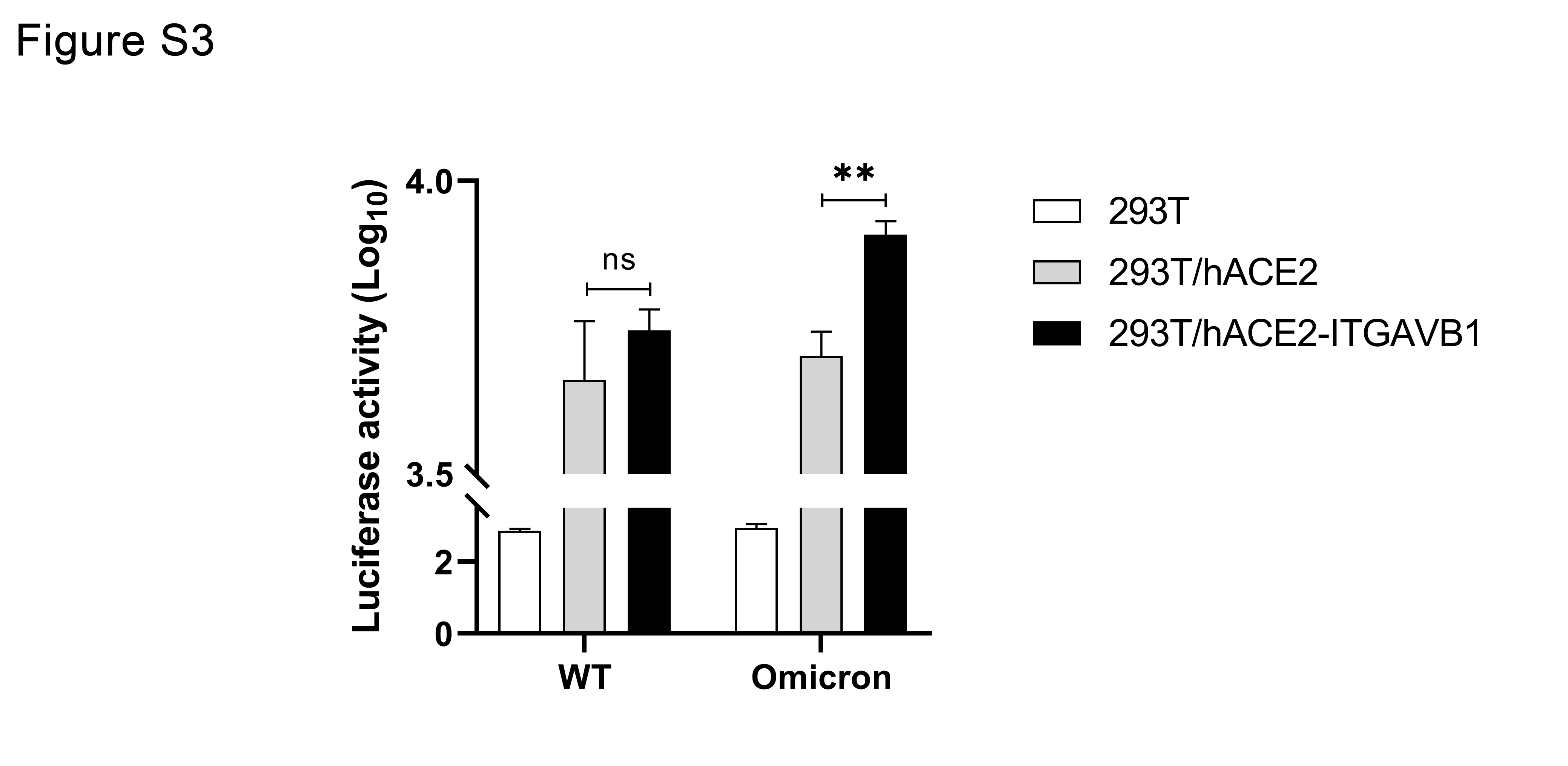
**

**Supplementary Figure 3 (SF3). The effect of integrin αvβ1 on the Omicron entry in 293T cell line.**

293T, 293T/hACE2 and 293T/hACE2-ITGAVB1 cells were infected with wild-type SARS-CoV-2 and Omicron, respectively. The viral entry efficiency was measured by luciferase activity assay at 72 h post infection. Significant difference between the groups were determined by two-tailed unpaired t test. **P < 0.01; Error bars indicate SD (n = 3).

Supplementary Table 1. Primers used for this study

| Gene | Primer | Sequence (5’-3’) |
| --- | --- | --- |
| ACE2 | F | GGGATCAGAGATCGGAAGAAGAAA |
|  | R | AGGAGGTCTGAACATCATCAGTG |
| GAPDH | F | CGGGAAGCTTGTGATCAATGG |
|  | R | GGCAGTGATGGCATGGACTG |
| Integrin α3 | F | TGTGGCTTGGAGTGACTGTG |
|  | R | TCATTGCCTCGCACGTAGC |
| Integrin α5 | F | GGCTTCAACTTAGACGCGGAG |
|  | R | TGGCTGGTATTAGCCTTGGGT |
| Integrin α-2β | F | GATGAGACCCGAAATGTAGGC |
|  | R | GTCTTTTCTAGGACGTTCCAGTG |
| Integrin αV | F | ATCTGTGAGGTCGAAACAGGA |
|  | R | TGGAGCATACTCAACAGTCTTTG |
| Integrin αM | F | GCCTTGACCTTATGTCATGGG |
|  | R | CCTGTGCTGTAGTCGCACT |
| Integrin αL | F | TGCTTATCATCATCACGGATGG |
|  | R | CTCTCCTTGGTCTGAAAATGCT |
| Integrin β1 | F | CCTACTTCTGCACGATGTGATG |
|  | R | CCTTTGCTACGGTTGGTTACATT |
| Integrin β2 | F | TGCGTCCTCTCTCAGGAGTG |
|  | R | GGTCCATGATGTCGTCAGCC |
| Integrin β3 | F | GTGACCTGAAGGAGAATCTGC |
|  | R | CCGGAGTGCAATCCTCTGG |
| Integrin β5 | F | TCTCGGTGTGATCTGAGGG |
|  | R | TGGCGAACCTGTAGCTGGA |
| Integrin β6 | F | TCCATCTGGAGTTGGCGAAAG |
|  | R | TCTGTCTGCCTACACTGAGAG |
| Integrin β8 | F | ACCAGGAGAAGTGTCTATCCAG |
|  | R | CCAAGACGAAAGTCACGGGA |

Supplementary Table 2. Antibodies used in this study

| ID | Source | Catalog | Application |
| --- | --- | --- | --- |
| Anti-Integrin β1 | Santa Cruz Biotechnology | sc-59829 | WB (1:200),  IF (1 ug/1x10^6 cells) |
| Anti-Integrin αV | Santa Cruz Biotechnology | sc-376156 | WB (1:100),  IF (1:50) |
| Anti-ACE2 | Proteintech | 21115-1-AP | WB (1:500-2000),  IF (1:50-500) |
| Anti-HIV1 p24 antibody | Abcam | ab63913 | WB (1:2500) |
| Anti-SARS-CoV-2 spike glycoprotein antibody- Coronavirus | Abcam | ab272504 | WB (1μg/mL) |
| Anti-GAPDH | Proteintech | 60004-1-Ig | WB (1:2000-1:12000） |
| Mouse IgG | Beyotime | A7028 | FC (50μg/mL) |
| Rabbit IgG | Beyotime | A7016 | FC (50μg/mL) |
| F(ab')2-Goat anti-Mouse IgG (H+L) Secondary Antibody, APC, eBioscience™ | Invitrogen | 17-4010-82 | FC (1:200) |
| Goat Anti-Rabbit IgG H&L (APC) Pre-adsorbed | Abcam | ab130805 | FC (1:100) |
| Peroxidase-AffiniPure Goat Anti-Mouse IgG (H+L) | Jackson | 115-035-003 | WB (1:5000) |
| Peroxidase-AffiniPure Goat Anti-Rabbit IgG (H+L) | Jackson | 115-035-003 | WB (1:5000) |
